# Cell-Type Targeted NF-kappaB Inhibition for the Treatment of Inflammatory Diseases

**DOI:** 10.3390/cells9071627

**Published:** 2020-07-06

**Authors:** Bettina Sehnert, Harald Burkhardt, Stefan Dübel, Reinhard E. Voll

**Affiliations:** 1 Department of Rheumatology and Clinical Immunology, Medical Center—University of Freiburg, Faculty of Medicine, University of Freiburg, 79098 Freiburg, Germany; 2 Division of Rheumatology, University Hospital Frankfurt, Goethe University, and Branch for Translational Medicine and Pharmacology TMP, Fraunhofer Institute for Molecular Biology and Applied Ecology IME, 60590 Frankfurt am Main, Germany; Harald.Burkhardt@kgu.de; 3 Institute of Biochemistry and Biotechnology, Technical University Braunschweig, 38106 Braunschweig, Germany; s.duebel@tu-bs.de

**Keywords:** NF-kappaB, autoimmune disease, inflammation, signaling pathways, cell type-specific regulation, sneaking ligand

## Abstract

Deregulated NF-k activation is not only involved in cancer but also contributes to the pathogenesis of chronic inflammatory diseases like rheumatoid arthritis (RA) and multiple sclerosis (MS). Ideally, therapeutic NF-KappaB inhibition should only take place in those cell types that are involved in disease pathogenesis to maintain physiological cell functions in all other cells. In contrast, unselective NF-kappaB inhibition in all cells results in multiple adverse effects, a major hindrance in drug development. Hitherto, various substances exist to inhibit different steps of NF-kappaB signaling. However, powerful tools for cell-type specific NF-kappaB inhibition are not yet established. Here, we review the role of NF-kappaB in inflammatory diseases, current strategies for drug delivery and NF-kappaB inhibition and point out the “sneaking ligand” approach. Sneaking ligand fusion proteins (SLFPs) are recombinant proteins with modular architecture consisting of three domains. The prototype SLC1 binds specifically to the activated endothelium and blocks canonical NF-kappaB activation. In vivo, SLC1 attenuated clinical and histological signs of experimental arthritides. The SLFP architecture allows an easy exchange of binding and effector domains and represents an attractive approach to study disease-relevant biological targets in a broad range of diseases. In vivo, SLFP treatment might increase therapeutic efficacy while minimizing adverse effects.

## 1. Introduction

The nuclear transcription factor-kappaB (NF-kappaB) is known for its crucial role during immune and inflammatory responses, cell growth, survival and development [1,2,3]. An aberrant NF-kappaB activation is not only associated to cancer development [4,5], but also contributes to the pathogenesis of chronic inflammatory diseases like rheumatoid arthritis (RA) and multiple sclerosis (MS). Herein, NF-kappaB dependent expression of pro-inflammatory cytokines, adhesion molecules, matrix degrading enzymes (MMPs) and other mediators play critical roles in initiation and perpetuation of chronic inflammation [6,7,8,9,10,11].

So far, an increasing number of studies have revealed the importance of NF-kappaB in inflammatory diseases and supports the concept of an NF-kappaB blockade for therapeutic interventions [12,13,14,15,16]. However, an ubiquitous pharmacologic NF-kappaB inhibition in every cell type might increase the risk of disturbing NF-kappa activity in its essential contribution to maintain homeostatic cellular pathways, for instance in immune cells and in the liver [17,18,19,20]. Further, NF-kappaB plays a critical role in resolution of inflammation which might also be compromised by systemic NF-kappaB inhibition [21,22]. To dampen NF-kappaB activity in a cell type-specific manner and to reduce adverse effects in clinical applications, pioneering approaches must be developed. This review provides an overview on the role of NF-kappaB in inflammatory diseases, on general strategies for drug delivery and NF-kappaB inhibition with a focus on the “sneaking ligand” (SL) approach as a promising strategy to improve the benefit risk ratio of a therapeutic targeting of the NF-kappaB pathway. The sneaking ligand approach represents a tool for cell-type specific targeting of signaling pathways and has been demonstrated to be applicable for a selective NF-kappaB inhibition in the cytokine-activated endothelium [23].

## 2. Relevance of NF-kappaB in Chronic Inflammatory Diseases

### 2.1. NF-kappaB Signaling

During the last decades, numerous publications and reviews highlighted the structure and function of individual components of the canonical or non-canonical (alternative) NF-kappaB cascade [2,24,25,26,27]. However, the specific relevance of canonical versus non-canonical NF-kappaB signaling in certain cell types contributing to the pathogenesis of immune-mediated inflammatory has not been fully uncovered. Considering the review of Noort and collegues [28] who described the “good ” and the “bad” sides of non-canonical NF-kappaB signaling in the context of RA, it might be appropriate not to confine the development of NF-kappaB inhibitors to the canonical pathway which is activated in cells expressing receptors for pro-inflammatory molecules like IL-1, TNF-alpha or LPS. Accordingly, we will briefly describe both signaling pathways first.

In mammals, five proteins termed p65 (RelA), RelB, c-Rel, p50 (NF-kappaB 1; and its precursor p105) and p52 (NF-kappaB 2; and its precursor p100) belong to the NF-kappaB family. These kappaB proteins possess a Rel-homology-domain (RHD) allowing DNA binding, dimerization, and nuclear translocation, whereas only p65, RelB and c-Rel exhibit a transcriptional activation domain (TAD) for gene activation. NF-kappaB members are capable to form any combination of homo- or heterodimers, however, not every theoretically possible dimer combination could be demonstrated to occur in vivo. Almost all cell types contain p50/p65 dimers, in addition, a physiological relevance of p50/p50, p50/c-Rel and p52/RelB dimers has been described [29,30,31,32].

In resting cells, p50/p65 heterodimers which are linked with the canonical pathway are retained in the cytoplasm due to their association to typical IkappaB proteins (IkappaB-alpha and IkappaB-beta) [30]. Signaling via IL-1, TNF-alpha and LPS and others leads to the formation and phosphorylation of the IKK complex which consists of the catalytic subunits IKK1 and IKK2 and the regulatory subunit NEMO. Following IkappaB-alpha phosphorylation through IKK subunits, IkappaB-alpha gets ubiquitinylated and subsequently degraded by the proteasome. Thereafter, the nuclear localization sequence (NLS) of p65 is exposed and shuttling of p50/p65 dimers into the nucleus is initiated [29,30,33]. Dimers of p50/p65 bind to promoters in chromosomal regions that harbor target genes encoding a variety of pro-inflammatory cytokines (IL-1, TNF-alpha, IL-6), adhesion molecules like E-selectin, VCAM-1, ICAM-1, and P-selectin as well as chemokines like MCP-1 and IL-8 in primary endothelial cells [32,34,35].

**Figure 1:**
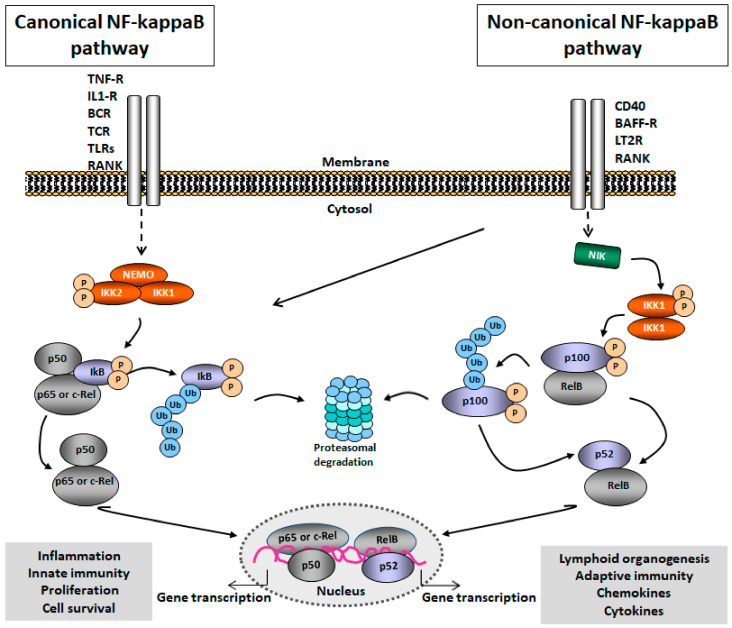
Nuclear factor-kappaB (NF-kappaB) signaling pathway. The canonical pathway (left) is activated through different receptors including TNF-receptor (TNF-R), IL-1 receptor (IL-1R), B-cell receptor (BCR), T-cell receptor (TCR), Toll-like receptor, RANK, and CD40. IKK/NEMO-induced phosphorylation of IkappB-alpha leads to nuclear translocation of NF-kappaB heterodimers (p50/p65, p50/c-Rel) and transcriptional activation of target genes. The non-canonical pathway (right) is activated through CD40, B-cell-activating factor receptor (BAFF-R), lymphotoxin beta receptor (LT2R) and RANK. Processing of p100 leads to the translocation of RelB/p52 heterodimers into the nucleus and activates target-gene transcription.

NF-kappaB signaling can also be initiated through stimuli like B-cell-activating factor (BAFF), CD40L, lymphotoxin beta (LTbeta) and receptor activator of NF-kappaB ligand (RANKL). These stimuli activate the non-canonical pathway which is independent of IKKbeta and NEMO. Instead, NF-kappaB inducing kinase (NIK) and IKKalpha regulate the processing of precursor p100 to p52 resulting in translocation of p52/RelB heterodimers into the nucleus. The non-canonical pathway has been shown to also play a major role the innate and adaptive immune responses [25] besides its well established critical importance for lymphoid organogenesis, B cell maturation and osteoclast differentiation (Figure 1).

A deregulated NF-kappaB activation is implicated to contribute to the pathogenesis of chronic immune-mediated diseases including RA and MS, asthma, inflammatory bowel disease and others [8,10,36,37,38,39]. In this review, we will focus on relevant research findings that address the role of NF-kappaB in RA and MS.

### 2.2. Role of NF-kappaB in RA

RA is a complex chronic inflammatory joint disease. The etiopathogenesis of RA is not yet fully understood. Genetic polymorphisms, epigenetic modifications and alterations of the intestinal microbiome in the context of environmental and life-style factors such as smoking, obesity, and nutrition habits, but also viral and bacterial infections contribute to the risk of RA development [40,41,42,43]. Synovial inflammation represents a hallmark of RA provoked by extravasation of various inflammatory cell types into the joints. A complex cellular network of T and B cells, macrophages, neutrophils, synovial fibroblasts, mast cells, endothelial cells and others, triggers the production of cytokines and other paracrine mediators that contribute as amplifying and disease perpetuating factors to drive the inflammatory process into systemic manifestations and chronicity. A local consequence of cytokine activation in the joint compartment is the accumulation of lymphocytes and myelomonocytic cells that upon their extravasation in the subintimal layer of the synovium contribute to the formation of a hyperplastic aggressively growing so called pannus tissue that can lead to irreversible damage of cartilage and bone [43,44,45].

**Figure 2:**
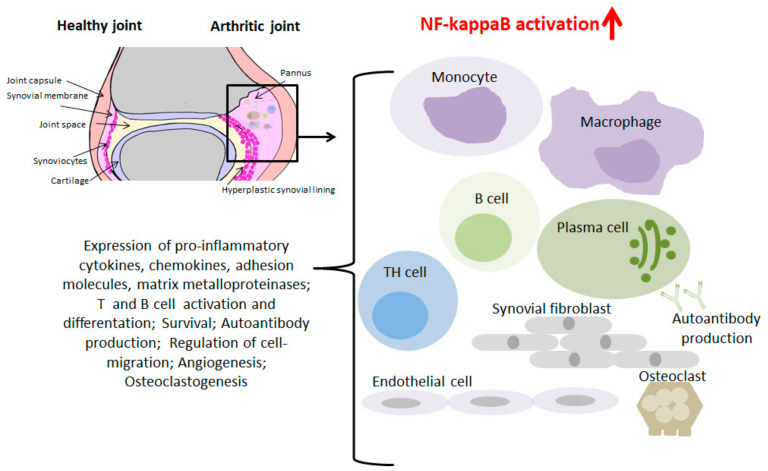
Role of NF-kappaB in RA. A Healthy and an arthritic joint is presented. Monocytes/macrophages, TH cells, B cells, plasma cells, synovial fibrobasts, endothelial cells, and osteoclasts contribute to RA pathogenesis. Induction of NF-kappaB target genes promotes inflammation leading to cartilage and bone destruction.

During recent years, extensive studies of biopsy material from RA patients, cell cultures and experimental models in rodents have been performed to gain insight into the functional role of NF-kappaB proteins and IKKs in RA pathogenesis. The synovial tissue of healthy subjects consists of a thin intima formed by 1–2 layers of cells consisting of cells with lineage characteristics of macrophages and probably fibroblasts. Under inflammatory conditions, monocytes, neutrophils, T and B cells migrate from circulation into the joint [46,47] (Figure 2).

Initial immunohistochemical studies demonstrated the presence of activated NF-kappaB in the inflamed synovial tissue of RA patients. In this study, tissue sections of synovitis patients were stained with a monoclonal antibody that specifically binds the nuclear localization signal (NLS) of p65. The NLS region in p50 and p65 gets unmasked after dissociation of IkappaB from the complex—which is induced by activators like IL-1, TNF-alpha and reactive oxygen intermediates (ROS). Afterwards NF-kappaB is translocated to the nucleus and activates the expression of many pro-inflammatory genes [48]. Active NF-kappaB was already detectable at early stages of RA especially in cells of the synovial microvasculature [49]. Immunohistochemical detectability of p50/p65 heterodimers was also demonstrated for CD14^+^ cells in RA synovial tissue emphasizing the importance of NF-kappaB for the upregulated expression of macrophage-derived cytokines in the rheumatoid synovium [50]. Collagen-induced arthritis (CIA) represents a well-established experimental murine model featuring characteristics of human RA including inflammation, pannus formation, cartilage and bone loss [51]. Studies of Han and colleagues suggested a link between metalloproteinase gene expression in the course of CIA and a preceding increase of NF-kappaB and AP-1 activation in the joints that was already detectable prior to arthritis onset [52]. Furthermore, an investigation in the experimental model of streptococcal-cell wall induced (SCW) arthritis in rats demonstrated that pharmacological inhibition of NF-kappaB improved clinical signs of arthritis associated with the induction of a profoundly enhanced apoptosis rate in the synovium [9]. Accordingly, the results uncover the protective anti-apoptotic properties conferred by an activated NF-kappaB pathway to synovial cells, thereby emphasizing its role as a critical link between inflammation and synovial hyperplasia.

Joint inflammation is accompanied by the production of pro-inflammatory cytokines like IL-1, IL-6 and TNF-alpha which are transcriptionally regulated by NF-kappaB. In turn, these cytokines can activate a variety of cells to contribute to joint pathology, such as T cells and fibroblasts that have been demonstrated to express different pro-inflammatory cytokines in an NF-kappaB-dependent manner. Thereby, IL-1/IL-6/IL-23-driven T helper (TH)17 differentiation followed by IL-17 production aggravates disease progression and induces chronic destructive arthritis [32,45,53,54].

Furthermore, the canonical and non-canonical NF-kappaB pathways can be induced by the receptor activator of NF-kappaB ligand (RANKL), which is expressed by a variety of cells in bone and bone marrow. Accordingly, NF-kappaB activity mediated by RANK/RANKL is involved in osteoclastogenesis thereby promoting bone loss in RA [55,56,57].

Elevated B-cell-activating factor (BAFF) levels have been identified in RA patients [58]. BAFF, a member of the family of TNF-like cytokines, is secreted by monocyte/macrophages, dendritic cells, neutrophils, stromal cells, and activated T cells in response to cytokine stimulation by TNF-alpha, IFN-gamma, and TGF-beta. Soluble BAFF binds to three receptors—BAFF-R (BAFF-R/BR3), transmembrane activator and calcium-modulating cyclophilin ligand interactor (TACI) and B cell maturation antigen (BCMA which activates canonical and non-canonical NF-kappaB signaling [59,60,61]. Among others, BAFF triggers the differentiation of T effector- and autoantibody-producing plasma cell populations, thereby contributing to pathogenesis in RA [58,62,63].

Earlier studies characterized the role of IKK2 in TNF-α-mediated NF-kappaB activation. Primary fibroblast-like synoviocytes from RA patients were transfected with a dominant negative IKK2 mutant that prevented TNF-α-mediated NF-kappaB nuclear translocation in contrast to transfections with a dominant negative IKK1 [64]. The potential role of IKK2 as key regulator in inflammation was subsequently addressed. Intraarticular transfer of an adenoviral construct encoding the IKK2 wild-type gene induced a joint inflammation in rodents suggesting IKK2 as an attractive target for pharmacological treatment of arthritis [65].

### 2.3. Role of NF-KappaB in MS

**Figure 3:**
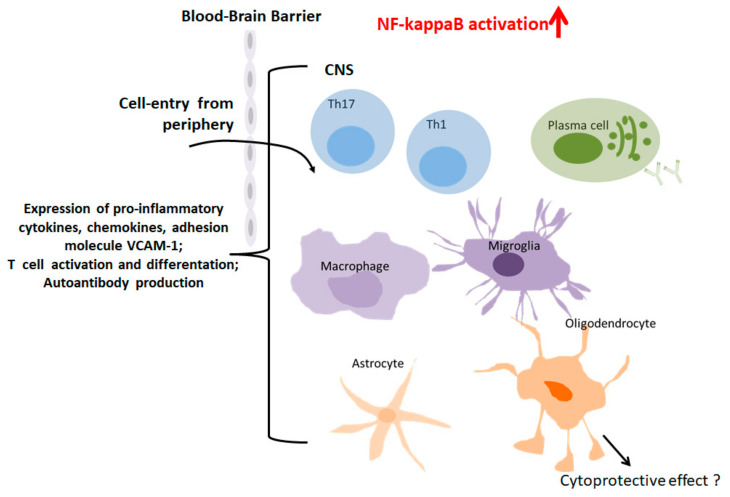
Role of NF-kappaB in MS. Macrophages/migroglia, TH cells, B cells, plasma cells, astrocytes and oligodendrocytes contribute to MS pathogenesis. Induction of NF-kappaB target genes promotes neuroinflammation, demyelination and neurodegeneration.

In 1868 the famous neurologist, Jean-Martin Charcot, lectured for the first time about the features of MS and coined its name [66]. MS is a chronic neuroinflammatory disease affecting the brain and spinal cord especially of young adults in Northern America and Europe [67]. Demyelination in the central nervous system (CNS) is a central hallmark in MS pathology and linked to complex interactions of CNS-specific CD4^+^ TH1 and TH17 cells, CD8^+^ effector T cells, microglia/macrophages and antibody-secreting plasma cells (Figure 3) [67,68].

The contribution of NF-kappaB in the development of this multifaceted neuroinflammatory disease is emphasized in many publications and reviews. Readers with a specific interest in the general role of NF-kappaB in MS are referred to the review of McGuire and colleagues [11]. Thus, the activation of the canonical NF-kappaB pathway induces the expression of nuclear transcription factors such as retinoic acid-related orphan receptor gamma t (RORgamma-T) in CNS-specific CD4^+^TH1 and CD4^+^TH17 cells. Subsequently, the differentiated T helper cells express the pro-inflammatory cytokine GM-CSF which seems to be a key regulator of the effector phase in neuroinflammation [69,70].

Experimental autoimmune encephalomyelitis (EAE) induced in susceptible mice by immunization with myelin-specific peptides in complete Freund’s adjuvant (CFA) or by the passive transfer of encephalitogenic T cells, is one of the most frequently used murine models for relapsing MS [71]. In EAE, IKK2 is essential for the expansion of autoreactive T cells so that either its deficiency in genetically engineered mice or its pharmacological inhibition in wild-type animals lead to diminished clinical signs of the experimental neuroinflammatory disease [72]. Moreover, CNS-restricted ablation of NEMO or IKK2 but not IKK1 ameliorated EAE pathology [73].

The impact on non-canonical NF-kappaB activation in T cells has not been fully elucidated. NF-kappaB-inducing kinase (NIK)–related signaling is known to regulate B-cell maturation and lymphoid organogenesis [25]. Mice lacking NIK are, however, resistant to EAE induction and exhibit a disturbed TH17 differentiation. These data suggest an essential role of NIK-signaling in T cells. Moreover, Jin et al. provided experimental evidence supporting an NIK-mediated synergistic induction of STAT3 tyrosine phosphorylation by TCR and IL-6R signals [74].

However, many questions remain open regarding myeloid-NF-kappaB activation in neuroinflammatory diseases. A previous study corroborates the hypothesis that constitutive activity of NF-kappaB in myeloid cells drives pathogenicity of monocytes and macrophages during autoimmune neuroinflammation. Conditional knockout-mice for IkappaB-alpha in myeloid cells exhibited a more severe clinical course compared to age-matched controls [75]. The important role of myeloid-cell derived NF-kappaB in neuroinflammation such as EAE was supported by a subsequent study. Herein, the authors demonstrated that IKK2-mediated inflammatory myeloid cell activation exacerbates experimental autoimmune encephalomyelitis by potentiating TH1/TH17 cell activation and decreased permeability of the blood brain barrier (BBB) indicating myeloid-IKK2 as a potential therapeutic target for treatment of MS [76]. The contribution of NF-kappaB activation in astrocytes to MS pathogenesis has also been investigated [11,77]. Transgenic inhibition of astroglial NF-kappaB attenuated EAE by suppressing chronic CNS inflammation and also prevented optic nerve damage and retinal ganglion cell loss in experimental optic neuritis [78,79]. Contrary to the characterized pro-inflammatory actions of NF-kappaB, also beneficial cytoprotective effects attributed to differential pathway activations have been observed in certain CNS cell-types such as oligodendrocytes. This fact has to be considered with regard to potential therapeutic interventions [80].

All these data illustrate the relevance of individual NF-kappaB proteins and IKKs in chronic inflammatory diseases such as RA and MS. Bearing in mind that NF-kappaB might also contribute to resolution of inflammation and exerts protective effects in certain cell types or tissues, a cell-selective guided delivery of NF-kappaB inhibitors might have great potential for clinical applications. So far, several different approaches have been developed to deliver substances into the cell’s interior to modulate effector function which will be reviewed in the next sections with the focus on strategies for NF-kappaB inhibition.

## 3. Delivery of Substances into the Cell’s Interior

Safeguarding of an efficient transport mechanism into the interior cell compartment is a requirement for a pharmacological agent whose therapeutic action on a signaling pathway requires a direct and selective binding to a specific intracellular ligand. Substantial industrial efforts were made to address the delivery of effector substances into the cell’s interior, the development of small molecule inhibitors, peptides, or liposomal-based drugs. Several FDA-approved drugs in liposomal formulations are on the market for indications like infections and cancer [81,82]. A major challenge represents the import of natural or synthetic molecules of high molecular weight or genetic material due to their physicochemical properties that render them unsuitable for crossing the lipid bilayer of the plasma membranes [83,84].

**Figure 4:**
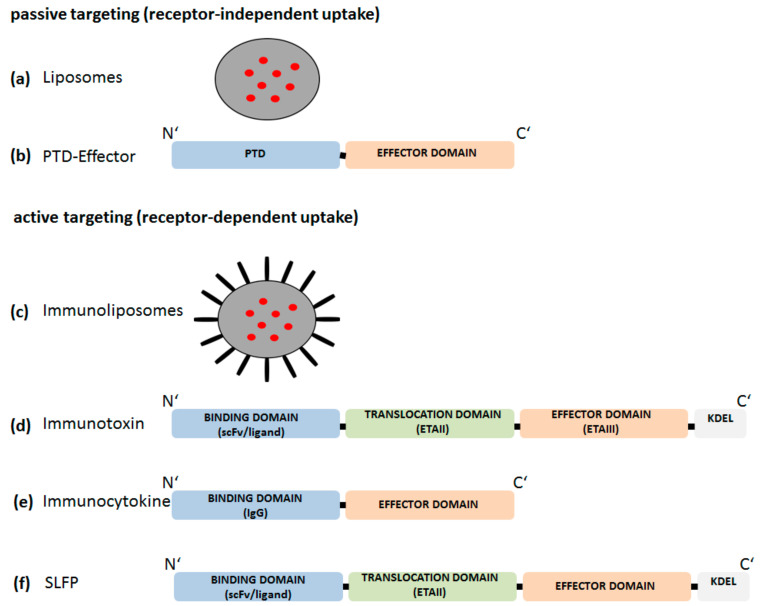
Tools for “passive” or “active” targeting. (**a**) non-specific uptake (passive targeting) of liposomes (grey circles) followed by intracellular release of encapsulated substances (red circles). (**b**) non-specific uptake of an effector molecule coupled to a protein transduction domain (PTD). (**c**) specific uptake (active targeting) of immunoliposomes (IP). Whole antibodies, ligands, or single chain variable fragments (scFv) (black bars) are coupled to the surface of liposomes (grey circles). IP release their encapsulated substances (red circles) after receptor-mediated uptake. (**d**) Recombinant modular proteins consisting of a specific binding domain (ligand or scFv), a translocation domain (ETAII) and an effector domain for cell killing (ETAIII). The KDEL motif is included for Golgi to endoplasmic reticulum (ER) transport. (**e**) Immunocytokines contain an IgG binding domain and an effector domain. (**f**) Sneaking ligand fusion proteins (SLFPs) are recombinant modular proteins consisting of a specific binding domain (ligand or scFv), the ETAII translocation domain (ETAII) and an effector domain for intracellular modulation of signaling pathways.

Mechanisms by which a drug is delivered to their site of action can be classified into “passive” or “active” targeting. In Figure 4 we illustrate schematically different approaches of “passive” or “active” targeting.

### 3.1. Passive Targeting

Liposomes, but also microspheres or nanoparticles are known to release their loaded proteins, synthetic substance, or DNA/RNA fragments into the cell after passive diffusion (Figure 4a). Accordingly, the release of the effector molecules is not restricted to a specific cell type. Liposomes are composed of natural materials and therefore offer the advantage of being nontoxic and biodegradable. However, high production costs, low solubility and short half-lives of liposomes are main disadvantages. Additionally, encapsulation of high molecular weight molecules tends to be a major limitation [85,86,87,88]. Moreover, liposomal-based drug delivery approaches might induce adverse immune phenomena such as complement activation related pseudo allergy (CARPA) and therapeutic failures due to PEG (poly(ethylene glycol)-related immunogenicity [89].

Membrane crossing of impermeable molecules can also be facilitated by protein transduction domains (PTDs). PTDs of various origins have been described and are also termed as cell-permeable proteins (CPPs) [90,91]. For instance, the targeted intracellular delivery of peptides/proteins incapable of cell membrane permeation can be improved by the aminoterminal fusion to either the 86-amino acid residues long basic protein region of the human immunodeficiency virus type 1 (HIV-1) TAT protein or the PTD deduced from the *Drosophila* homeoprotein antennapedia (Antp) (amino acid sequence residues 47–57) (Figure 4b) [92,93]. Despite great research efforts, the uptake mechanism of CPPs is not yet fully understood, considering inter alia passive delivery, endocytosis-mediated or inverted micelle-mediated delivery [93,94,95]. Furthermore, to date, no CPP-based candidate has yet received the status of an FDA-approved drug for clinical application. Currently, there are two clinical trials registered which investigate a cell-penetrating prototypic compound (p28) targeting p53 ubiquitination for treatment of solid cancer [96,97,98].

### 3.2. Active Targeting

First attempts for cell-specific uptake of substances were made by the development of immunoliposomes/-particles. Whole antibodies, scFv or ligands were attached to liposome/particle surfaces to achieve specificity for selective binding to receptor structures expressed on the surface of the target cells. After receptor-mediated endocytosis, the encapsulated molecules are released into the cell and can achieve their pharmacodynamic effect via interaction with their respective intracellular target structures (Figure 4c). Immunoliposomes/particles are already approved in cancer therapy [99,100]. However, nanotechnology-based drug delivery systems have also disadvantaged that might impede application in vivo. Inefficient rates of release of active substance into the cytoplasm, or low stability limit their therapeutic use [101].

An alternative approach for cell-type specific delivery of an effector molecule is based on the architecture of the three-domain structure of natural toxins like *Pseudomonas* Exotoxin A (PE or ETA) [102,103,104,105] and is utilized in immunotoxins (IT), whereby the binding domain ETAIa is replaced by a cell type/receptor-specific ligand (scFv or ligand) (Figure 4d) [106,107,108]. For ETA, the intoxication pathway has not yet been fully elucidated but is suggested to consist of the following sequence of events: Receptor-mediated endocytosis of ETA leads to formation of early and late endosomes. Within the endocytic pathway, ETA is proteolytically cleaved by the endoprotease furin at Arg279 which is localized in the translocation domain (ETAII) resulting in two fragments. One fragment consists of parts of domain II, domain Ib and the ADP ribosyltransferase domain and is subsequently transported from the Golgi apparatus to the endoplasmatic reticulum (ER) in a retrograde manner. This Golgi-ER retrograde transport of ETA is mediated by a C’-terminal motif REDL element binding to the KDEL-receptor [109]. The catalytic ADP ribosyltransferase domain is subsequently transported into the cytoplasm possibly via the Section 61 translocon and promptly inactivates elongation factor 2 (EF2) by ADP ribosylation which inhibits protein synthesis and kills the cell [105,109,110,111]. The application of immunotoxins is not restricted to cancer therapy, but also suggested as a tool to eliminate cell types contributing to inflammatory disease conditions. One example might be a CD64-based immunotoxin to eliminate activated macrophages [112]. In recent years, macrophage research emphasized the phenotypic differentiation of macrophages into M1 (inflammatory) and M2 (anti-inflammatory) subsets under polarizing conditions, for instance during the course of chronic inflammatory diseases [113,114,115]. A recent review about M1/M2 macrophages and RA discussed the contribution of M1/M2 subsets in blood and synovial tissue to pathogenesis of RA. The authors conclude that a strict classical division into M1 and M2 subsets and a comparison in different samples such as blood, synovial fluid and synovial membrane of RA patients might be questionable. Further, membrane surface markers that predicts a M1 or M2 phenotype were mostly not coherent with the currently observed function status of the cell (anti- or pro-inflammatory) [116]. Further research effort is needed to find useable M1/M2 markers for in vivo investigations as most of them are not congruent to markers found in vitro [117].

Recently, a novel therapeutic concept based on recombinant proteins was introduced. These engineered immunocytokines composed of tissue specific binding domains linked to an effector domain and were also named as “armed antibody” (Figure 4e) [118]. The “armed antibody” DEKAVIL includes the human antibody F8, specific for the extra-domain A of fibronectin linked to the human anti-inflammatory cytokine IL-10. F8 exhibits a strong affinity to cells from synovial biopsies and was shown to inhibit the progression of collagen-induced arthritis [118]. The phase IB clinical trial for DEKAVIL showed first promising results on safety and reduction of disease activity in RA patients [119]. Immunocytokines might be a novel therapeutic option targeting immunomodulatory cytokines to the site of inflammation or tumor growth [120,121].

Despite a variety of already established strategies there is still unmet need for unique multi-domain fusion proteins as tools for modulating intracellular signaling pathways in a cell-type and activation status dependent manner, e.g., for targeted treatment approaches to inhibit NF-kappaB. We have developed so called sneaking ligand fusion proteins (SLFP). SLPF’s are structurally related to immunotoxins and comprise a cell-type and activation status-specific binding domain, the ETAII translocation domain, and an effector domain for targeting intracellular molecules (Figure 4f) [23,122,123].

## 4. Approaches for NF-kappaB Inhibition

A series of studies using transgenic and knockout mice were performed to decipher critical elements of the NF-kappaB pathway that are involved in driving the development of pathologic phenotypes. Moreover, transgenic and knockout technology can promote the understanding of NF-kappaB in various cell types and can help to discover strategies for therapeutic manipulation [124,125].

Over time, extensive research has focused on the relevance of NF-kappaB in the immune system, cancer and inflammation, which has led to the development and characterization of hundreds of NF-kappaB inhibitors targeting distinct key steps in the NF-kappaB cascade. An extensive variety of NF-kappaB inhibitors including chemicals, synthetic compounds, peptides, proteins (cellular, viral, bacterial and fungal), natural products, metals, metabolites, but also physical conditions are known (for review see Gupta et al. and Gilmore et al. [126,127]). For further details see reviews of strategies for therapeutic inhibition of NF-kappaB [128,129,130,131]. The development of inhibitors that target the IKK complex, block NF-kappaB nuclear translocation and DNA binding, but also inhibitors that block NF-kappaB activation by protein phosphatases, methyltransferases or p65 acetylation has remained a dynamic area of research and drug development over time. Approaches to inhibit IkappaB ubiquitinylation and proteasomal degradation also seem to be powerful tools and are currently of great interest for drug development. Some agents for proteasomal inhibition are now approved for clinical use in the treatment of malignancies. Based on data from experimental models and case reports, a first clinical trial was conducted to investigate the effect of bortezomib, a proteasome inhibitor, in antibody-mediated autoimmune diseases [132,133]. Furthermore, the management of inflammatory diseases includes the application of nonsteroidal anti-inflammatory drugs (NSAID’s), glucocorticoids (GC’s) and conventional disease modifying drugs (cDMARD’s) such as methotrexate, often in combination with biologicals or JAK inhibitors. Here, we focus on potential new anti-inflammatory drugs, small molecule inhibitors, peptides for IKK targeting/disruption and substances for proteasomal inhibition, agents, that are already approved or might be useful for NF-kappaB inhibition in chronic inflammatory diseases like arthritis.

Persistent joint inflammation causes irreversible damage of cartilage and bone associated with reduced mobility that may finally lead to disability. RA patients are commonly treated with NSAIDs, GC’s and immunomodulatory so-called disease modifying antirheumatic drugs (DMARD’s).

### 4.1. NF-kappaB Inhibition by NSAIDs and GC’s

The prototypic NSAID discovered more than 100 years ago is Aspirin, an acetyl derivative of salicylic acid [134]. It was shown that Aspirin inhibits prostaglandin (PG) synthesis by inhibiting cyclooxygenases 1 and 2 (Cox-1 and Cox-2), enzymes that transform arachidonic acid into PG’s [135,136]. PG’s are inflammatory mediators that contribute to reinforcement and amplification of inflammatory processes in the joint during arthritis [137]. However, growing evidence from a variety of studies on the mode of action revealed that the observed beneficial effect of Aspirin in inflammatory diseases is not confined to its inhibition of COX-activity by acetylation. The postulate for PG-independent anti-inflammatory pharmacodynamics activities lead to the elucidation of the property of Aspirin to act as a competitive inhibitor of the ATP-binding site in IKK2. As a consequence, IkappaB phosphorylation is blocked and NF-kappaB activation is inhibited [129,138,139,140]. Moreover, COX-2 was identified as one of the target genes upregulated in response to NF-kappaB supporting a strong impact of the latter on an enhanced PG production during inflammation [141]. Several studies suggest that salicylate-mediated NF-kappaB inhibition leads to a downregulated expression of adhesion molecules like VCAM-1 and ICAM-1 in endothelial cells resulting in a reduced extravasation of leukocytes to the sites of inflammation [142]. Moreover, it was shown that Aspirin is capable of reducing IL-12 expression that subsequently leads to a compromised development into TH1 cells, a T effector cell subset critically involved in promoting inflammatory arthritis [143]. For Ibuprofen, another traditional NSAID, but also the selectively COX-2 blocking agent Celecoxib, a partial dependency of their anti-inflammatory effect on PGE2 production could be ascribed to NF-kappaB inhibition resulting in a reduced COX-2 expression [140,144,145].

Upon their discovery in the 1940s, GCs have proven their high efficiency in the treatment of inflammatory and autoimmune conditions and are accordingly ranked among the most powerful and frequently applied drugs in these indications. GCs including dexamethasone, prednisone and methylprednisolone have been reported to inhibit NF-kappaB activation. Various studies sought to discover the mode of action of GCs and finally concluded that GCs can not only inhibit IKK activity and DNA binding of NF-kappaB, but also transactivation [20,146]. Accordingly, GC application suppresses the expression of NF-kappaB target genes such as IL-6, which is a strong activator of IL-6R-mediated JAK-STAT signaling, another signal transduction pathway implicated in RA progression [147]. However, besides the beneficial effect of NSAIDs and GC’s, their usage is limited due to severe side effects. NSAID’s may cause liver damage, impairment of renal function, gastroduodenal ulcerations and bleedings, and are not recommendable for patients with a risk profile for NSAID-toxicity [148]. The therapeutic decision for the use of GCs has to balance their benefits of proved high efficacy and low costs with the increased risk to develop severe side effects especially associated with long-term application including infections, development of high blood pressure, cardiovascular events, increase of body weight, osteoporosis and others [147,149].

### 4.2. Other Inhibitors of NF-kappaB

Further investigations identified agents for inhibition of IkappaB degradation, translocation and p65 acetylation, but also a large number of IKK interfering molecules were developed and intensively characterized [127].

Proteasome inhibitors such as bortezomib are small molecules which have the capacity to inhibit NF-kappaB activity by blocking proteasomal degradation of IkappaB alpha and other regulatory and misfolded proteins. Bortezomib induced amelioration of clinical manifestations in lupus mice and even in an initial “proof of concept” open label study in SLE patients by eliminating autoreactive plasma cells [133,150,151]. However, also in CIA, clinical severity and histological manifestations were significantly improved upon bortezomib treatment [152].

Small cell-permeable peptides like SN50, that contains the nuclear localizing sequence of p50, but also double stranded oligodeoxynucleotides (ODN) with a consensus NF-kappaB sequence exert anti-inflammatory effects by blocking NF-kappaB nuclear translocation in a mouse model of acute inflammation [153,154]. However, SN50 showed reduced NF-kappaB selectivity as it also blocks other transcription factors including AP-1, STAT1 and NFAT [155].

Another approach to modulate NF-kappaB activity is to intervene with the activity of the IKK complex. Most of the IKK inhibitors generally interact with the conserved adenosine triphosphate (ATP)–binding site of IKK molecules and exert anti-inflammatory properties [126,131]. The IKK inhibitor sc-514 competes with ATP binding to IKK2 and represents a selective IKK2 inhibitor which reduced cytokine gene expression by inhibiting IkappaB-alpha phosphorylation/degradation, affecting NF-kappa B nuclear import/export as well as the phosphorylation and transactivation of p65 in cytokine-activated synovial fibroblasts derived from RA patients [156]. In contrast, BMS345541 acts as an allosteric inhibitor for IKK1 and IKK2, but with a 10-fold higher selectivity towards IKK2. This cell-permeable quinoxaline compound exhibits beneficial effects in joint inflammation and cartilage destruction in CIA [157,158].

IKK complex assembly represents a key step in NF-kappaB activation. In 2000, May and colleagues identified a NEMO-binding peptide (NBP) that blocks the association of NEMO with IKK2. NBP consists of the carboxyl-terminal segment of IKK2 and was linked to a PTD that facilitates the entry of NBP into cells. Consequently, cytokine-induced NF-kappaB activation and NF-kappaB gene expression was inhibited. Hence, the Antp-linked NBP represents a sophisticated inhibitor of cytokine-induced NF-kappaB activation, while leaving the basal NF-kappaB activity intact [14,159].

Taken together, a large number of NF-kappaB inhibitors were extensively investigated but with respect to clinical application, most of them still do not fulfill the challenging requirements regarding pharmacokinetics and/or benefit/risk profiles for a promising candidate to enter late stages of a cost-intensive clinical development program [131]. A systemic NF-kappaB inhibition bears the risk for the development of severe adverse effect especially with respect to long-term treatment [18]. Hence, the development of strategies for cell-type specific NF-kappaB inhibition might shift the benefit/risk judgement to a more favorable ratio for application in inflammatory diseases like RA and MS. In this respect the so called “sneaking ligand” -(SL) approach [23] provides a promising tool to improve our understanding of the differential impact of NF-kappaB activation in certain cell types on the inflammatory diseases. The prototypic sophisticated instrument for cell-type and activation state specific NF-kappaB inhibition is described below.

## 5. The “Sneaking Ligand” (SL) Approach for NF-kappaB Inhibition

### 5.1. Modular Structure and Mode of Action of an Endothelium-Specific NF-KappaB Inhibitor

SLFPs are recombinant proteins that comprise of three domains: first, the N-terminal cell surface binding domain, second, the ETAII translocation domain and third, the C’-terminal effector peptide interacting with its cytoplasmic ligand to modulate signaling pathways. The general composition has already been depicted in Figure 4f. Similar to ETA, a KDEL motif is included at the C’-terminus of SLFPs to ensure the initiation of a Golgi-ER retrograde transport via the KDEL-receptor and release of the effector domain into the cytoplasm [110].

Biologically active recombinant proteins are mostly produced in *E. coli*. Low cost and high yields are the main advantages of bacterial expression [160]. A limitation of this system is the lack of appropriate post-translational modification and the expression of large proteins which can be achieved in mammalian cells [161,162]. In order to reach high yield expression, insertion of SL-DNA constructs in different vector systems and expression in different E.coli strains is mostly indispensable to identify the best vector/host system [160]. Affinity tags (e.g., His_6_-tag or Strep-tagII) are introduced into the constructs to facilitate purification and detection [163]. According to our experience, ligation of the multi-modular synthetic SLFP gene into the pAK400 plasmid using SfiI restriction sites and expression in *E. coli* HB2151 seems to be the most powerful combination among others and results in high yield expression [122,164].

Transmigration of inflammatory cells through the cytokine-activated endothelium is a hallmark in inflammatory diseases [165]. As earlier mentioned, pro-inflammatory cytokines like IL-1 and TNF-alpha trigger activation of the canonical NF-kappaB pathway in pathologic inflammatory conditions and induce the expression of adhesion molecules like E-selectin, ICAM-1, VCAM-1, and P-selectin as well as chemokines like MCP-1 and IL-8 [34,35,165,166]. An early sequelae upon cytokine activation of the vascular endothelium is the induction of E-selectin supported leukocyte rolling initiating a sequence of events that finally lead to transendothelial migration [167,168].

**Figure 5:**
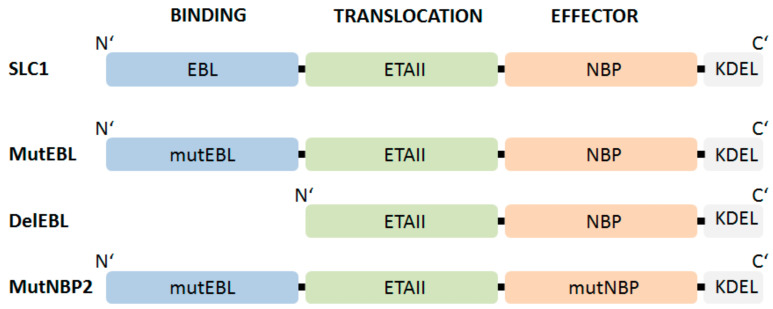
Schematic representation of functional and non-functional SLFPs to investigate E-selectin specific-NF-kappaB inhibition. SLC1 consists of an E-selectin binding motif named EBL (E-selectin binding ligand): EBL encodes 3 repeats of an E-selectin specific-peptide (AF 10166, aa: DITWDQLWDLMK) [169] connected with S_4_G–linkers; ETAII: translocation domain of Pseudomonas exotoxin A domain II (aa 253–364) [170] and NBP (Nemo-binding peptide) encompassing amino acid 644-756 of IKK2 [14,159]. MutEBL: EBL was replaced by 3 repeats of a scrambled peptide (aa: WKLDTLDMIQD) [169]. DelEBL: EBL-deleted form of SLC1. MutNBP2: interaction region of NEMO corresponds to a scrambled peptide (aa: DLAWQTFLTES). The respective domains were connected via (S_4_G)_2_ linkers [23].

The critical role of NF-kappaB in cytokine-induced activation of endothelial cells and its impact on arthritis pathogenesis could be elucidated by studies of the pharmacologic modulation of these processes by SLC1, an SLFP that specifically binds to E-selectin [23]. A prerequisite for this approach was the availability of E-selectin as a target structure selectively upregulated in a cell-type specific fashion upon induction by cytokines released under inflammatory conditions. Figure 5 depicts the three-modular structured functional SLC1 and respective non-functional SL variants that we have used in our studies as controls.

**Figure 6:**
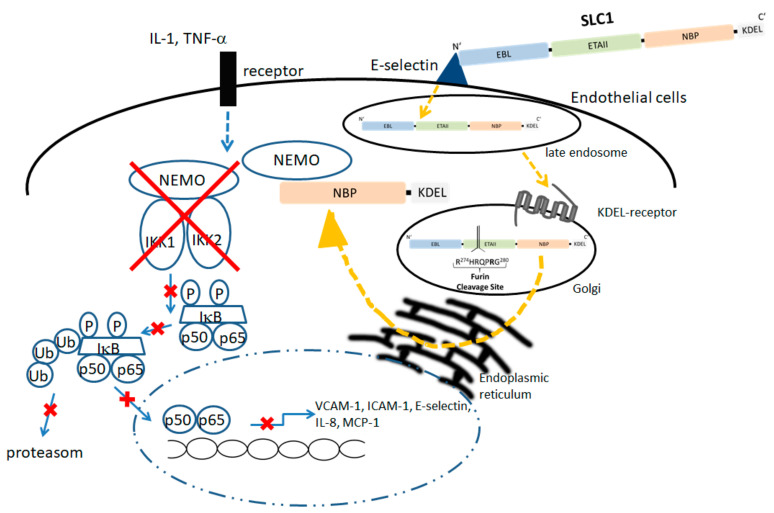
The SLC1 is taken up by the endothelial cell via E-selectin-mediated endocytosis and reaches upon engulfment a late endosomal compartment; via the KDEL-receptor bearing Golgi, SLC1 is passed through the ER in a retrograde fashion. In the ER, the ETAII is cleaved by furin and the C-terminal NBP element is released into the cytoplasm. Subsequently the recombinant NBP traps NEMO and leads to inhibition of IKK complex assembly (right panel). As SLC1 treatment results in a blockade of IKK assembly, subsequent steps of cytokine-induced NF-kappaB activation and finally translocation of p50/p65 dimers into the nucleus are blocked in the endothelial cells (blocked steps are marked with red crosses).

In SLFPs, the binding ligand should fulfill criteria of high affinity binding to the respective complementary structure on the surface of the target cell. Natural ligands, single chain Fv or phage display derived peptides can be used. For the design of SLC1 a high affinity ligand for E-selectin was identified by Martens et al. [169]. Accordingly, a triple repeat of this E-selectin binding motif (AF10166, amino acid sequence: DITWDQLWDLMK) derived from a peptide phage display library was introduced as binding domain (EBL; E-selectin binding ligand) in our prototypic SLC1 [23,122,169]. The translocation domain comprises full-length ETAII (amino acid sequence 253–364 of ETA) [170] and initiates the transport of NBP (amino acids 644–756 from IKK2) into the cytoplasm to interfere with IKK complex assembly. To characterize the functionality of each domain a group of non-functional fusion proteins were generated. MutEBL consists of a scrambled peptide (amino acid sequence: WKLDTLDMIQD) [169], whereas the E-selectin binding element was deleted in DelEBL. The ability of SLC1 to inhibit NF-kappaB activation was further compared to MutNBP2 in which the critical NEMO interaction site was changed to a scrambled form (DLAWQTFLTES) (Figure 5). Once NBP is released into the cytoplasm, the peptide binds to the regulatory subunit of the IkappaB kinase complex. Figure 6 illustrates E-selectin-mediated uptake of SLC1, processing and NEMO interaction (Figure 6, right side) thereby inhibiting cytokine-stimulated canonical NF-kappaB activation and blocking the expression of multiple pro-inflammatory target genes (Figure 6, left side) [34,35].

### 5.2. Biological Effects of SLC1 In Vitro and Vivo

In vitro experiments demonstrated specific uptake of SLC1 in E-selectin expressing cells followed by inhibition of cytokine-induced translocation of p50 and p65 into the nucleus. Transient transfection of an NF-kappaΒ luciferase reporter gene was used as an indicator tool to characterize the impact of SLC1 on transcriptional activity of NF-kappaB in wild-type Chinese hamster ovary (CHO) cells (E-selectin negative) or E-selectin expressing CHO cells [171,172]. SLC1 reduced transcriptional activity of NF-kappaB significantly in E-selectin expressing cells, but not in wildtype cells. Further, adhesion and transmigration assay revealed the relevance of NF-kappaB activation in cytokine-activated endothelial cells and the capacity of SLC1 to block these NF-kappaB-dependent processes efficiently [23].

**Figure 7:**
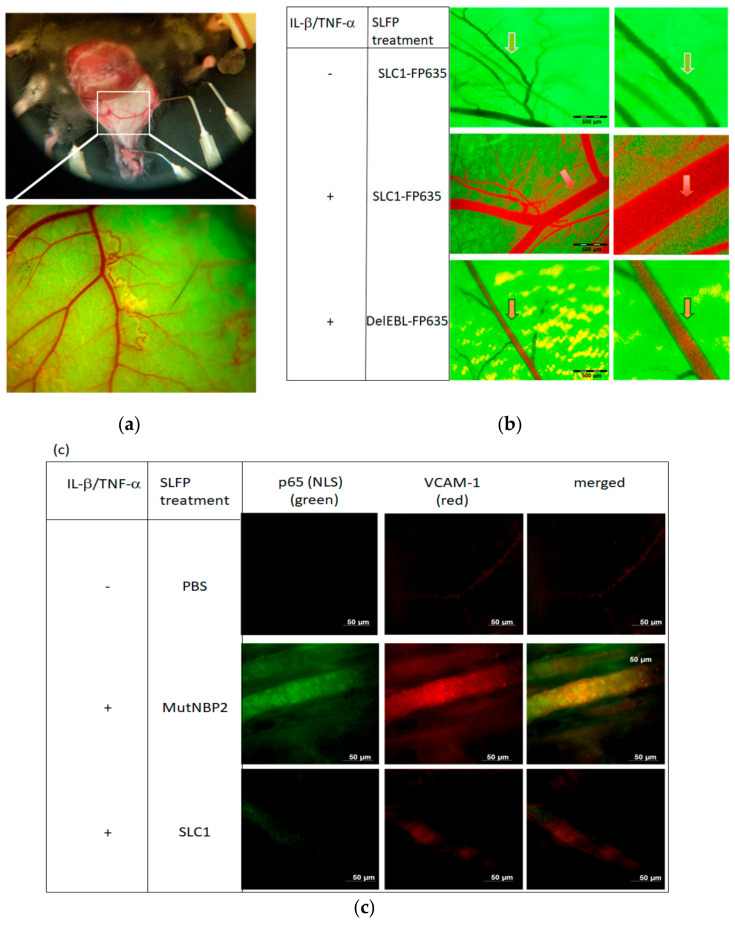
In vivo imaging to visualize SLC1 binding and inhibition of NF-kappaB activation. (**a**) Skin flap preparation of GFP expressing transgenic mice under anaesthesia. In vivo images of the vasculature in a selected area of the tissue are depicted at different magnifications ranging from ×1.6 to ×16 and a field of view ranging from 6.9 to 0.69 mm. (**b**) Visualization of vessels in a non-activated state (upper row) exhibits the GFP-background fluorescence but no staining by the systemically administered red-fluorescent SLC1-FP635. Mice receiving a local cytokine-challenge (lL-1β/TNF-α) showed an intense orange staining (arrow) of the vascular endothelium (middle row) after systemic SLC1-FP635 treatment, whereas in DelEBL treated mice only a green fluorescence of the endothelial border is present (lower row). (**c**) Whole-mount immunofluorescence stainings of cytokine-activated mouse skin. Mice were treated intraperitoneally (i.p.) with PBS, MutNBP2 or SLC1. Whole mount was stained with an anti-NF-kappaB-(NLS) p65 antibody (green) detecting nuclear p65 and an anti-VCAM-1 antibody (red). NLS-p65 exposure and VCAM-1 expression is not detectable in unactivated (-IL-1β/TNF-α ) PBS-treated mice (upper row). NLS-p65 and VCAM-1 is detectable in activated (+ IL-1β/+TNF-α ) mice treated with the control MutNBP2 (middle row). SLC1 treatment suppresses NF-kappa activation in activated mice. Here the NLS-p65 region is not detectable accompanied by reduced endothelial VCAM-1 expression (lower row) (Figures from [23], kindly provided by PNAS).

Subsequent studies were initiated to evaluate if cell type specificity and effectivity of NF-kappaB inhibition using SLC1 or SLFPs in general can be observed also therapeutic settings in vivo [23]. First, in vivo imaging [173,174] was used to prove the selective binding of SLC1 to cytokine-activated but not to resting endothelium in mice. An epigastric vein in a skin flap of live transgenic green fluorescent protein (GFP) expressing mice was selected as the region of interest for detection of SLC1 effects on cytokine activation of the vascular endothelium (Figure 7a).

Systemic injection of red-infrared labelled SLC1 into GFP mice did not alter the green fluorescent appearance of the non-activated vasculature (Figure 7b upper panel). However, in mice receiving a local IL-1 beta/TNF-alpha challenge, we observed a redistribution of SLC1 (red) to the vessel wall resulting in a strongly stained orange border of the endothelial lining. This observation reflects specific SLC1 binding to E-selectin, which was upregulated upon cytokine-treatment. (Figure 4b middle panel). In contrast, DelEBL did not accumulate to the cytokine-activated endothelial cells whereas only a green fluorescent lining layer appears (Figure 4b, lower panel) [23].

The impact of SLC1 in NF-kappaB translocation was further investigated by whole mount immunofluorescence microscopy of cytokine-induced mouse skin. Similar to the analysis of Gilston and colleagues [49], we used an anti-p65 (NLS) antibody to examine NF-kappa activation. No anti-p65 (NLS) antibody staining was observed in SLC1 treated mice indicating that NBP interferes with IKK complex assembly followed by an inhibition of IkappaB alpha degradation (Figure 7c, lower row, left staining). In contrast, mice receiving a scrambled form of NBP (MutNBP2) showed an intensive anti-p65(NLS) staining revealing the exposure of the p65-NLS region by NF-kappaB activation (middle row, left staining). Uninduced mice receiving PBS did not show any staining (upper row) demonstrating a non-activated vasculature. As adhesion molecules like VCAM-1 are transcriptionally regulated by NF-kappaB, we were interested in VCAM-1 expression in cytokine-activated mouse skin. Therefore, sections were counterstained for VCAM-1. Functional SLC1 (lower row, middle staining) but not MutNBP2-treated mice (middle row, middle staining) caused a reduction of endothelial VCAM-1 expression suggesting a preventive effect of SLC1 on cell migration into areas of ongoing inflammation.

Thus, our data indicate that an E-selectin specific NF-kappaB inhibitor is able to block NF-kappaB activation and suppresses the expression of VCAM-1 as an adhesion molecule with functional impact on cell migration in vivo [23].

A main goal of the development of cell-type specific NF-kappaB inhibition is its potential for translation into therapeutic application. Therefore, the therapeutic efficacy of SLC1 was assessed in experimental mouse models of arthritis [23,123]. One frequently used model that reflects the effector phase of human RA, is the K/BxN serum-transfer induced arthritis model (STIA) [175]. Established STIA shares a variety of features with human RA, including polyarticular manifestations, leukocyte infiltration, pannus formation, synovitis, as well as cartilage and bone erosions [176], thereby rendering it a suitable experimental disease model for the further evaluation of the anti-arthritic potential of SLC1. In STIA, the onset of an acute polyarthritis is induced in mice receiving systemic injections of serum containing antibodies recognizing glucose-6 phosphoisomerase (anti-G_6_PI), an ubiquitous antigen that accumulates in joint cartilage due to high affinity interactions with glycosaminoglycans of the cartilage matrix. The formation of anti-G_6_PI-autoantibodies/G6PI immune complexes at the cartilage surface induces complement-mediated attraction and Fc-receptor-activation of neutrophils and macrophages and provoke the release of pro-inflammatory cytokines (IL-1 and TNF-α) [175]. Our study showed that repeated systemic injections of SLC1 significantly reduced clinical signs of arthritis in the treatment group compared to cohorts of mice that either received the MutNBP2 variant or PBS for control. Comparison of treatment efficacy between SLC1 and Antp-NBP revealed an equipotent therapeutic effect at a 20-fold lower molar concentration of SLC1 supporting the advantage of the cell-targeted approach of NF-kappaB inhibition. Histological analysis demonstrated a decrease of synovial inflammation, as well as reduced cartilage and bone loss in SLC1-treated STIA mice [123,175].

### 5.3. Outlook for the Application of SCL1

Thus, our data indicate that an E-selectin specific NF-kappaB inhibitor can block joint inflammation thereby protecting cartilage and bone against structural damage. Based on published data on shared pathophysiological pathways we hypothesize that inflammation in septic shock [154], disseminated intravascular coagulation (DIC) [154], atherogenesis [155] or tumor neoangiogenesis [156,157] might be susceptible to attenuation by E-selectin specific NF-kappaB inhibition as well.

However, the future development of the SLC1 approach for therapeutic application in other immune mediated diseases will require additional work, e.g., on the production procedure including quality control, stability, pharmacokinetics as well as sophisticated in vivo imaging studies. Preliminary SDS PAGE/Western blot data demonstrate stability of SLC1 and its respective non-functional control constructs during long-term storage for up to 1 year at 4 °C with only very minor degradation and aggregation products. Moreover, additional data provides evidence that biologic activity of SLC1 in vitro and in vivo is kept unaltered for storage periods of ~4 weeks. A significant therapeutic benefit of a substance also depends on its long-lasting effect. Preferentially treatment should not require daily dosing. However, in RA-treatment, some biologics such as anakinra or etanercept exert their beneficial activity only upon continuous daily or weekly subcutaneous (sc.) injections [177]. Despite occuring injection site reactions severe side effects leading to treatment discontinuation are rather infrequent. For inhibition of endothelial NF-kappaB activation mice received 3 injections of SLC1 within a time of 24 h simultaneously with arthritis induction whereas the therapeutic effect lasted approximately 8 days. The SLC1 effect on the improvement of clinical signs have not been investigated upon treatment initiation in already established arthritis or following regimens with a reduced injection frequency, e.g., a single dose. However, our data provide clear evidence that additional injections of SLC1 at day 2, 4 and 5 does not result in a more pronounced reduction of disease severity demonstrating the fast effect of SLC1 mediated NF-kappaB inhibition in activated endothelial cells [23].

## 6. Conclusions

To date, no gold standard for the treatment of chronic inflammatory diseases has been identified. In this respect NF-kappaB represents a very attractive potential therapeutic target due to its central importance for a plethora of pathogenic inflammatory pathways. However, systemic NF-kappaB inhibitors may cause serious adverse events especially those associated with severe immunosuppression [18,19]. In this respect and compared to the already described NF-kappaB inhibition strategies, the SL-approach has the advantage that it combines the strong therapeutic potential of blocking a crucial transcriptional activator of inflammation with a risk reduction achieved by restricting NF-kappaB blockade in a cell-type specific and activation state dependent manner. SLC1 is the prototype of a recombinant NF-kappaB inhibitor selectively targeting the cytokine-activated endothelium that exerts strong anti-inflammatory effects in models of murine arthritis [23]. Due to the results, we postulate that also other inflammatory disease conditions for instance septic shock [178], DIC [178], atherogenesis [179] or tumor neoangiogenesis [180,181] might benefit from an adapted treatment strategy based on the prototypic SLC1. Even though the development of the SL-approach makes an important contribution to the field of cell-selective modulation of intracellular signaling pathways, SLFP’s have to be refined further in order to reduce immunogenicity to render them applicable for the treatment of autoimmune diseases and cancer, otherwise repeated applications may lead to anti-drug antibodies and loss of efficacy or even immune complex-mediated disease. Such strategies aim at using natural ligands and shortening the ETAII domain as recently described for a construct just containing the furin cleavage site for ER processing [110]. Furthermore, it is known that some ligands for receptor binding require their location at the C-’terminus of SLFPs to exert sufficient binding affinity. Therefore, SLFPs with a C’-terminal encoded binding domain linked to an N’-terminal effector domain has to be developed. A recombinant IT comprising a C’-terminal binding domain was described by Heisler et al. [182]. In summary, cell type-specific NF-kappaB-inhibition may be a highly useful treatment strategy in multiple inflammatory and potentially malignant diseases.
